# National Health Policy and factors predicting its implementation at the local level in Nepal: an exploratory cross-sectional study

**DOI:** 10.3389/fpubh.2025.1592213

**Published:** 2025-07-04

**Authors:** Dipendra Khadka, Zhang Xinyi, Zhang Mengjie, Rajan Bhusal, Chichen Zhang

**Affiliations:** 1 School of Public Health, Southern Medical University, Guangzhou, Guangdong, China; 2 School of Health Management, Southern Medical University, Guangzhou, Guangdong, China; 3 Key Laboratory of Philosophy and Social Sciences of Colleges and Universities in Guangdong Province for Collaborative Innovation of Health Management Policy and Precision Health Service, Guangzhou, Guangdong, China; 4 School of International Education, Southern Medical University, Guangzhou, Guangdong, China; 5 Hospital and Rehabilitation Centre for the disabled children (HRDC), Banepa, Nepal

**Keywords:** national health, healthcare workers, health policy, Nepal, public health

## Abstract

**Introduction:**

Nepal’s National Health Policy (NHP), periodically revised since 1991, aims to enhance citizens’ health and well-being within the federal democratic framework established in line with the Constitution of Nepal 2015. This study evaluated the implementation status, challenges, and opportunities of the NHP at the local level in Nepal.

**Materials and methods:**

A descriptive cross-sectional study was conducted in the Lumbini province among 166 health workers selected through simple random sampling. Data were collected from October 10 to November 15, 2024, using a structured questionnaire developed in Nepali through literature review and expert consultations. Field researchers administered the questionnaire. The final tool assessed awareness, challenges, and factors influencing the National Health Policy implementation. Descriptive statistics and inferential statistics using binary logistic regression were used for analysis.

**Results:**

The study revealed moderate awareness of NHP among health workers, with 60.2% somewhat familiar with the policy. Major implementation challenges included inadequate infrastructure (62.0%), limited access to skilled healthcare professionals (56.0%), and insufficient financial resources (54.2%). Regular use of technology significantly increased the likelihood of positive NHP implementation outcomes (OR: 5.448, 95% CI: 1.988–14.926, *p* = 0.001). Age (OR: 0.919, 95% CI: 0.880–0.961, *p* < 0.001) and years of experience (OR: 0.934, 95% CI: 0.895–0.975, *p* = 0.002) were significantly associated with attitudes toward NHP implementation. While 65.7% of respondents were somewhat aware of local NHP initiatives, 86.8% expressed the need for additional training and resources.

**Conclusion:**

The study found moderate awareness of the National Health Policy among health workers, with key challenges including inadequate infrastructure, limited skilled professionals, and insufficient funding. Regular use of technology significantly improved implementation outcomes, while younger age and less experience were linked to more positive attitudes. Despite some awareness of local initiatives, most participants stressed the need for additional training and resources to enhance policy execution.

## Introduction

Nepal’s NHP, which was first implemented in 1991 and has been periodically revised, with the latest update in 2019, plays a pivotal role in enhancing the health and well-being of its citizens (1, 2). The adoption of Nepal’s new constitution in 2015 marked a significant shift from a unitary government to a federal system, establishing Nepal as a federal democratic republic with three levels of autonomous government: the federal level, seven provinces, and 753 local governments, each with an elected assembly (3, 4). Local elections in late 2017 further solidified this transition, the first in two decades (5, 6), signaling the effective implementation of federalism in Nepal (6).

Nepal’s diverse topography and socioeconomic conditions pose unique challenges for its healthcare system, yet significant strides have been made in improving healthcare access and implementing the NHP (7). The NHP is at the core of these efforts, primarily focusing on ensuring equitable access to quality healthcare for all citizens, regardless of their geographic or socioeconomic status. However, questions remain about the policy’s effectiveness at the local level, which continues to be underrepresented in healthcare improvements. These issues have sparked ongoing research and debate (8).

At the same time, Nepal has been working toward implementing the Sustainable Development Goals (SDGs), coinciding with the federalization of the health sector (9). One of the key components of the NHP is developing an equity-focused health system based on the principles of Universal Health Coverage (UHC) (10). This includes health insurance programs, free basic healthcare services, transparent and accountable health services, and capacity-building initiatives outlined in the Nepal Health Sector Plan and its implementation plan (11, 12). With the support of UHC Partnership activities, some progress has been made in these areas (13).

Despite these successes, significant challenges persist in achieving equitable healthcare access, especially locally (14). Unequal service distribution, inadequate healthcare infrastructure, and limited resource allocation continue to hinder progress (15, 16). The rising burden of non-communicable diseases, mental health issues, and health concerns arising from natural disasters further complicate healthcare delivery (11). These factors have made it difficult to implement the NHP fully, underscoring the need for closer scrutiny of the policy’s effectiveness and identifying opportunities for improvement (17).

The NHP, through its various revisions, has set ambitious goals to tackle the country’s major health issues, including reducing maternal and child mortality, controlling communicable diseases, and preventive healthcare (18). However, the translation of these policy goals into measurable improvements at the local level has been inconsistent (19). Factors such as inadequate resource allocation, weak governance structures, and limited human capacity have impeded the full realization of the policy’s potential (20). This research aims to critically assess the implementation of the NHP at the local level in Nepal, focusing on the challenges and opportunities within the federal context. By examining the relationships and coordination between the federal, provincial, and local governments, this study seeks to identify key bottlenecks hindering effective policy implementation. Additionally, the research will explore the experiences of healthcare providers and community members to understand the lived realities of the NHP at the grassroots level.

## Materials and methods

### Study design and setting

This research employs a quantitative descriptive cross-sectional design to evaluate the implementation of Nepal’s NHP in Lumbini Province, a region encompassing around 5 million residents across 109 local administrative units. These units include four sub-metropolitan cities, 32 municipalities, and 73 rural municipalities, reflecting a socio-geographically diverse landscape ranging from urbanizing hubs to remote rural areas. This setting provides a strategic context to analyze decentralized health governance under federalism, enabling systematic comparisons of infrastructure, workforce distribution, financial allocations, and service coverage metrics across urban, semi-urban, and rural tiers. The cross-sectional approach facilitates a snapshot assessment of policy-driven outcomes and urban–rural disparities in health system performance, aligning with the province’s administrative and demographic complexities.

### Participants

This study employed a quantitative descriptive cross-sectional design to assess Nepal’s NHP implementation in Lumbini Province. Participants were selected through simple random sampling from a comprehensive roster of health workers across all 109 local administrative units (4 sub-metropolitan cities, 32 municipalities, 73 rural municipalities) to ensure representation of urban, semi-urban, and rural contexts. The sample size (*n* = 166) was calculated using the formula *n* = Z^2^ × p × (1 – p) / d^2^, with a 95% confidence level (*Z* = 1.96), a margin of error (*d* = 5%), and an assumed proportion (*p* = 87.2%) (21) of health workers familiar with similar policies, derived from a prior study. Inclusion criteria targeted health professionals directly involved in policy execution, including medical doctors, nursing staff, allied health workers (e.g., Health Assistants, lab technicians), pharmacists, and Public Health Inspectors/Officers (PHI/PHOs), spanning diverse career stages (0–15 + years of experience) and employment modalities (permanent, contract, temporary, volunteer). Exclusion criteria were implicit: individuals not engaged in frontline health service delivery or those outside the province’s administrative jurisdiction were excluded. The final sample achieved a 96.5% response rate, minimizing non-response bias and capturing perspectives across socio-geographically distinct tiers. Participants were recruited proportionally from urbanizing hubs and remote rural areas to reflect infrastructure, resource access, and governance disparities. This approach ensured a holistic evaluation of NHP implementation barriers and facilitators within the province’s decentralized health system.

### Measures

#### Independent variables

Likert-type questions were employed. Awareness of the NHP was measured on a 5-point scale, ranging from “not familiar at all” to “very familiar.” Participants were also asked to rate their understanding of the objectives and components of the NHP on a similar scale, from “not well at all” to “very well.” Additional data were gathered on the sources of NHP information, such as training sessions, official documents, workshops, or peer interactions. Awareness of NHP initiatives at the local level was assessed using a 5-point scale from “not aware at all” to “very aware.” To identify areas for improvement, participants rated their perceived need for further training or resources for NHP implementation on a scale from “strongly agree” to “strongly disagree.”

Next, participants’ perceptions of challenges and opportunities in implementing the NHP were explored. Challenges assessed included key factors such as healthcare access, the effectiveness of health insurance, regulation of medical products, and the profit- versus service-oriented approach in the health sector. These items were rated on scales appropriate to each challenge—for example, from “very low” to “very high” for healthcare access and from “not effective at all” to “extremely effective” for insurance effectiveness. Opportunities in NHP implementation were evaluated across areas such as responsibility sharing among different levels of government, the potential for increased public health awareness, advancements in healthcare technology and quality management, and the role of statistical data in decision-making. Each opportunity was rated using relevant scales to quantify participants’ perceptions. Finally, the study assessed factors influencing NHP implementation. Participants rated elements such as the clarity of NHP goals, the adequacy of financial resources, the influence of politics, and the competency of health personnel. These factors were evaluated on a scale ranging from “strongly agree” to “strongly disagree” to provide insight into perceived facilitators or barriers to effective policy implementation.

#### Dependent variables

The 5-point Likert scale responses regarding factors influencing the implementation of the NHP of ordinal scale data were aggregated and transformed into an interval scale. For analysis, the interval-scale data were dichotomized into two categories: “Agree” (combining “Strongly Agree” and “Agree”) and “Disagree” (combining “Neutral,” “Disagree,” and “Strongly Disagree”) using the SPSS software (22). Descriptive statistics, including means and standard deviations, were calculated for each aggregated statement to summarize the data. The normality of the transformed interval data was assessed using the Shapiro–Wilk test. The results indicated that the data met the assumption of normal distribution (23), justifying its use in further parametric analyses. Binary logistic regression was then employed to explore the relationship between independent variables—financial resources, political influences, health personnel competence, and public health expenditures—and the dichotomized dependent variable. The dependent variable categorized perceptions as “positive” or “negative” toward the implementation of the NHP (24) based on the mean cut-off.

### Data collection

The data collection took place between October 10 and November 15, 2024, using a structured questionnaire developed in Nepali. The questionnaire was designed based on a thorough review of relevant literature and consultations with subject matter experts, including national health policymakers and government advisors. A pilot test involving 10% of the sample (17 participants) from Kirtipur Municipality was conducted to assess its reliability and clarity, leading to minor revisions before its final implementation. Data collection was carried out by seven trained enumerators (four females and three males), all of whom were public health graduates with a sound understanding of Nepal’s health system. These enumerators underwent 3 days of rigorous training focused on ethical considerations, using the questionnaire, and maintaining consistency during data collection. Each interview lasted approximately 30–40 min, allowing adequate time to gather detailed participant responses.

The data was initially collected using paper-based questionnaires and later transferred to an electronic format using password-protected tablets to enhance accuracy and minimize transcription errors. The digitized data was uploaded daily to a secure, centralized server, ensuring immediate backup and protection against data loss. To maintain strict privacy, access to the data was limited to authorized members of the research team, and personal identifiers were removed during the data cleaning process to ensure anonymity. Confidentiality was prioritized throughout the study, with informed consent obtained from all participants before the interviews. Participants were assured that their responses would remain anonymous and used exclusively for research. The research followed the ethical guidelines of the Nepal Health Research Council (NHRC), adhering to national and international data privacy and confidentiality standards.

### Statistical analysis

The collected data were thoroughly checked for consistency and completeness before being entered into Microsoft Excel. Quantitative data were analyzed using descriptive statistics, such as means, standard deviations, frequencies, and percentages. Statistical analyses were conducted using the Statistical Package for the Social Sciences (SPSS) version 20 and Microsoft Excel. Binary logistic regression analysis was employed to identify significant associations, with variables having a *p*-value ≤ 0.05 considered statistically significant.

### Ethical considerations

The study obtained ethical approval from the Nepal Health Research Council (Ref. No: NHRC-024-722) and written informed consent from all participants. Maintaining participant confidentiality and anonymity was crucial, given the sensitive nature of some topics covered, such as perspectives on political influences and resource allocation challenges. This ethical approach ensured the participants’ protection and the data’s integrity.

## Results

The study participants included a diverse group in terms of age, gender, and professional background. Regarding age, 32.5% were under 30, 43.4% were between 30 and 40, 21.7% were 40–50, and 2.4% were over 50. Most participants were female (61.4%), with males making up 38.6%. Regarding professional categories, 49.4% were Allied Health Workers, 28.3% were Nursing Staff, 9.6% were Medical Doctors, 6.6% were Pharmacists, and 6.0% were Public Health Inspectors/Officers (PHI/PHO).

Regarding work experience, 23.5% had 0–5 years, 33.1% had 5–10 years, 16.3% had 10–15 years, and 27.1% had more than 15 years. Regarding employment status, 61.4% were permanently employed, 9.6% were on contract, 17.5% worked part-time or temporarily, and 11.4% were volunteers. Technology use at work showed that 71.1% used it regularly, 16.9% occasionally, and 12.0% rarely (detailed in a Supplementary material).

Access to healthcare resources was moderate for 41.6% and insufficient for 58.4% of participants. Regarding training on the NHP, 14.5% had received training, while 85.5% had not. Local government support for NHP implementation was reported by only 9.0% of participants, with 91.0% indicating a lack of support. Finally, sources of NHP information varied, with 36.7% learning from official documents, 32.5% from training sessions, 12.7% from colleagues or superiors, 10.2% from workshops or seminars, and 7.8% from other sources (Supplementary material).

Table 1 presents the awareness and understanding of the NHP among local health workers. Regarding familiarity with the NHP, 100 (60.2%) reported being somewhat familiar, 43 (25.9%) were very familiar, 14 (8.4%) were neutral, 8 (4.8%) were not very familiar, and 1 (0.6%) were not familiar at all. In terms of comprehensive understanding of the NHP’s objectives and components, 89 (53.6%) stated they understood moderately well, 30 (18.1%) understood very well, 24 (14.5%) were neutral, 21 (12.7%) did not understand very well, and 2 (1.2%) did not understand well at all. When asked about their primary source of information on the NHP, 61 (36.7%) cited official documents, 54 (32.5%) mentioned training sessions, 21 (12.7%) learned from colleagues or superiors, 17 (10.2%) referred to workshops or seminars, and 13 (7.8%) reported other sources. For awareness of NHP strategies and initiatives at the local level, 109 (65.7%) were somewhat aware, 18 (10.8%) were very aware, 20 (12.0%) were neutral, 16 (9.6%) were not very aware, and 3 (1.8%) were not aware at all. Finally, regarding the need for additional training or resources to enhance understanding of the NHP, 81 (48.8%) agreed, 63 (38.0%) strongly agreed, 19 (11.4%) were neutral, 2 (1.2%) disagreed, and 1 (0.6%) strongly disagreed.

**Table 1:** Awareness and understanding of the NHP among health workers at the local level.

Statement	Frequency (*N*)	Percent (%)
I am familiar with the National Health Policy (NHP).		
Not familiar at all	1	0.6%
Not very familiar	8	4.8%
Neutral	14	8.4%
Somewhat familiar	100	60.2%
Very familiar	43	25.9%
I have a comprehensive understanding of the key objectives and components of the NHP.		
Not well at all	2	1.2%
Not very well	21	12.7%
Neutral	24	14.5%
Moderately well	89	53.6%
Very well	30	18.1%
I primarily receive information about the NHP through training, official documents, workshops/seminars, and colleagues.		
Training sessions	54	32.5%
Official documents	61	36.7%
Workshops or seminars	17	10.2%
Colleagues or superiors	21	12.7%
Other	13	7.8%
I am aware of the strategies and initiatives outlined in the NHP being implemented at the local level.		
Very aware	18	10.8%
Somewhat aware	109	65.7%
Neutral	20	12.0%
Not very aware	16	9.6%
Not aware at all	3	1.8%
I feel the need for additional training or resources to enhance my understanding of the NHP.		
Strongly agree	63	38.0%
Agree	81	48.8%
Neutral	19	11.4%
Disagree	2	1.2%
Strongly disagree	1	0.6%

1 (Strongly disagree) to 5 (Strongly agree).

Table 2 outlines the challenges and opportunities for implementing the NHP in local communities. Access to healthcare services among citizens was rated as moderate by 110 (66.3%) of respondents, while 22 (13.3%) rated it high, 24 (14.5%) rated it low, 9 (5.4%) rated it very low, and 1 (0.6%) rated it very high. The effectiveness of health insurance implementation was rated slightly effective by 63 (38.0%), moderately effective by 61 (36.7%), very effective by 33 (19.9%), not effective at all by 6 (3.6%), and extremely effective by 3 (1.8%). Regarding challenges related to the regulation of medicine and medical products, 90 (54.2%) agreed that there were challenges, 43 (25.9%) remained neutral, 15 (9.0%) disagreed, 9 (5.4%) strongly disagreed, and 9 (5.4%) strongly agreed. Respondents perceived the health sector as equally profit- and service-oriented by 54 (32.5%), while 34 (20.5%) viewed it as mostly service-oriented, 34 (20.5%) as mostly profit-oriented, 34 (20.5%) as completely service-oriented, and 10 (6.0%) considered it completely profit-oriented. Responsibility sharing for health services at federal, state, and local levels was rated poorly by 61 (36.7%), adequately by 39 (23.5%), well by 45 (27.1%), very poorly by 17 (10.2%), and very well by 4 (2.4%). Opportunities to increase public awareness of health-related issues were rated good by 54 (32.5%), limited by 55 (33.1%), some by 44 (26.5%), none by 9 (5.4%), and excellent by 4 (2.4%). Advancements in information technologies, drugs, and equipment were observed slightly by 67 (40.4%), moderately by 66 (39.8%), not at all by 16 (9.6%), very much by 15 (9.0%), and extremely by 2 (1.2%). Emphasis on management and quality of health in policies and programs was rated slightly by 73 (44.0%), moderately by 71 (42.8%), very much by 14 (8.4%), not at all by 8 (4.8%), and extremely by none. Finally, the use of statistics in policymaking and decision-making processes was rated poor by 78 (47.0%), fair by 38 (22.9%), good by 28 (16.9%), very poor by 21 (12.7%), and excellent by 1 (0.6%).

**Table 2:** Challenges and opportunities for implementing the NHP in local communities in Nepal.

	Variables	Frequency (*N*)	Percent (%)
Challenges	Access to healthcare services among citizens		
	Very Low	9	5.4%
	Low	24	14.5%
	Moderate	110	66.3%
	High	22	13.3%
	Very High	1	0.6%
	Health insurance effectiveness in implementation		
	Not Effective at All	6	3.6%
	Slightly Effective	63	38.0%
	Moderately Effective	61	36.7%
	Very Effective	33	19.9%
	Extremely Effective	3	1.8%
	Medicine and medical products regulation challenges		
	Strongly Disagree	9	5.4%
	Disagree	15	9.0%
	Neutral	43	25.9%
	Agree	90	54.2%
	Strongly Agree	9	5.4%
	Profit-oriented health sector rather than service-oriented		
	Completely Profit-Oriented	10	6.0%
	Mostly Profit-Oriented	34	20.5%
	Equally Profit- and Service-Oriented	54	32.5%
	Mostly Service-Oriented	34	20.5%
	Completely Service-Oriented	34	20.5%
Opportunities	Responsibility sharing for health services at federal, state, and local levels		
	Very Poorly	17	10.2%
	Poorly	61	36.7%
	Adequately	39	23.5%
	Well	45	27.1%
	Very Well	4	2.4%
	Opportunities to increase public awareness of health-related issues		
	No Opportunities	9	5.4%
	Limited Opportunities	55	33.1%
	Some Opportunities	44	26.5%
	Good Opportunities	54	32.5%
	Excellent Opportunities	4	2.4%
	Observe advancements in new information technologies, drugs, and equipment		
	Not at All	16	9.6%
	Slightly	67	40.4%
	Moderately	66	39.8%
	Very Much	15	9.0%
	Extremely	2	1.2%
	Emphasis on health policies and programs in management and quality of health		
	Not at All	8	4.8%
	Slightly	73	44.0%
	Moderately	71	42.8%
	Very Much	14	8.4%
	Extremely	0	0.0%
	Rating of statistics use in policymaking and decision-making processes		
	Very Poor	21	12.7%
	Poor	78	47.0%
	Fair	38	22.9%
	Good	28	16.9%
	Excellent	1	0.6%

Regarding the main obstacles to providing free, quality basic health services in the local community, financial constraints were reported by 54.2% of participants. Limited access to skilled healthcare professionals affected 56.0% of respondents, while inadequate infrastructure and facilities were highlighted by 62.0%. High out-of-pocket healthcare costs were a concern for 29.5% of participants. Inefficient health insurance policies impacted 43.4% of respondents; although 97.0% reported no awareness, only 3.0% indicated experiencing awareness issues (detailed table on a Supplementary material).

Table 3 shows respondents’ perceptions of various aspects of implementing the NHP. A majority, 139 (45.8%), agreed that the objectives of the NHP are clear, while 100 (33.1%) were neutral, and 32 (10.8%) strongly agreed. A smaller portion, 30 (10.2%), disagreed or strongly disagreed with the clarity of the objectives. Regarding the understandability of the NHP goals, 170 (56.6%) found them understandable, with 78 (26.5%) remaining neutral and only 11 (3.6%) expressing disagreement. Regarding financial resources, 78 (26.5%) strongly agreed, and 114 (38.6%) agreed that insufficient financial resources exist for the NHP’s implementation. A significant portion, 91 (30.7%), remained neutral on this issue. When it came to the clarity of the financial resource distribution, 144 (48.8%) agreed that it was clear, while 75 (25.3%) were neutral, and 62 (21.1%) felt it was unclear. Concerning political influence, 83 (28.3%) believed that political influence had a positive effect on NHP implementation, while 108 (36.7%) agreed with this view. However, 18 (6.0%) disagreed, and 12 (4.2%) strongly disagreed. Regarding political interference negatively affecting the implementation, 87 (29.5%) strongly agreed, 114 (38.6%) agreed, and 73 (24.7%) were neutral, indicating a concern about political interference. Regarding health personnel competence, 114 (38.6%) agreed that personnel were sufficiently competent, but 41 (13.8%) disagreed or strongly disagreed, suggesting concerns about the workforce’s preparedness. A significant 136 (45.8%) felt a shortage of competent personnel to effectively implement the NHP, with 56 (18.7%) strongly agreeing. Regarding the allocation of public health expenses, 119 (40.4%) agreed that the budget was adequately allocated for healthcare needs, although 37 (12.7%) disagreed or strongly disagreed. Furthermore, 70 (23.5%) strongly agreed, and 136 (45.8%) agreed that public health expenses are insufficient, indicating dissatisfaction with the financial investment. Regarding the availability of resources for successful implementation, 136 (45.8%) agreed that sufficient resources were available, while 61 (20.5%) thought there was a shortage. Lastly, 127 (42.8%) of respondents agreed that their organization strongly supports the NHP, with 103 (34.9%) remaining neutral, and 139 (47%) felt that health workers actively contribute to its successful implementation.

**Table 3:** Factors influencing the implementation of the NHP.

Statement	Frequency (*N*)	Percent (%)	Mean	Std. deviation
The objectives outlined in the National Health Policy are communicated.	166	100	2.22	1.19
Strongly agree	18	10.8		
Agree	76	45.8		
Neutral	55	33.1		
Disagree	14	8.4		
Strongly disagree	3	1.8		
The goals of the National Health Policy are easily understandable.	166	100	2.22	1.14
Strongly agree	22	13.3		
Agree	94	56.6		
Neutral	44	26.5		
Disagree	3	1.8		
Strongly disagree	3	1.8		
The financial resources allocated for NHP implementation are sufficient.	166	100	2.13	1.19
Strongly agree	44	26.5		
Agree	64	38.6		
Neutral	51	30.7		
Disagree	6	3.6		
Strongly disagree	1	0.6		
There is a lack of clarity in the distribution of financial resources for the NHP.	166	100	2.20	1.15
Strongly agree	35	21.1		
Agree	81	48.8		
Neutral	42	25.3		
Disagree	6	3.6		
Strongly disagree	1	0.6		
Political influences positively contribute to the effective implementation of the NHP.	166	100	2.21	1.16
Strongly agree	47	28.3		
Agree	61	36.7		
Neutral	41	24.7		
Disagree	10	6.0		
Strongly disagree	7	4.2		
Political interference negatively impacts the successful execution of the NHP.	166	100	2.17	1.18
Strongly agree	49	29.5		
Agree	64	38.6		
Neutral	41	24.7		
Disagree	8	4.8		
Strongly disagree	3	1.8		
Health personnel are well-equipped and competent in implementing NHP strategies.	166	100	2.47	1.19
Strongly agree	28	16.9		
Agree	64	38.6		
Neutral	51	30.7		
Disagree	14	8.4		
Strongly disagree	9	5.4		
There is a shortage of competent health personnel to execute the NHP effectively.	166	100	2.33	1.18
Strongly agree	31	18.7		
Agree	76	45.8		
Neutral	34	20.5		
Disagree	23	13.9		
Strongly disagree	2	1.2		
Public health expenses for the NHP are appropriately allocated to address healthcare needs.	166	100	2.47	1.16
Strongly agree	28	16.9		
Agree	67	40.4		
Neutral	43	25.9		
Disagree	21	12.7		
Strongly disagree	7	4.2		
The current allocation of public health expenses is inadequate for the comprehensive implementation of the NHP.	166	100	2.17	1.18
Strongly agree	39	23.5		
Agree	76	45.8		
Neutral	38	22.9		
Disagree	10	6.0		
Strongly disagree	3	1.8		
There are ample human and material resources to support the successful implementation of the NHP.	166	100	2.48	1.16
Strongly agree	25	15.1		
Agree	76	45.8		
Neutral	34	20.5		
Disagree	22	13.3		
Strongly disagree	9	5.4		
Human and material resources are insufficient to meet the requirements of NHP initiatives.	166	100	2.13	1.18
Strongly agree	34	20.5		
Agree	92	55.4		
Neutral	27	16.3		
Disagree	11	6.6		
Strongly disagree	2	1.2		
The organization provides strong support for the implementation of NHP initiatives.	166	100	2.40	1.14
Strongly agree	23	13.9		
Agree	71	42.8		
Neutral	58	34.9		
Disagree	10	6.0		
Strongly disagree	4	2.4		
Individual health workers actively engage and contribute to the successful execution of the NHP.	166	100	2.34	1.18
Strongly agree	25	15.1		
Agree	78	47.0		
Neutral	46	27.7		
Disagree	15	9.0		
Strongly disagree	2	1.2		

1 (Strongly disagree) to 5 (Strongly agree).

Table 4 shows that the binary logistic regression analysis revealed that age was a significant predictor of the implementation of the NHP (CoR = 0.919, 95% CI [0.880–0.961], *p* < 0.001). Gender was not significantly associated, with males showing a CoR of 0.815 (95% CI [0.426–1.557], *p* = 0.536) compared to females. Profession categories showed no significant differences compared to the reference group (PHI/PHO/Pharmacists), with nursing staff (CoR = 0.667, 95% CI [0.167–2.666], *p* = 0.566), allied health workers (CoR = 0.943, 95% CI [0.303–2.932], *p* = 0.919), and medical doctors (CoR = 0.565, 95% CI [0.199–1.604], *p* = 0.283) all yielding non-significant results.

**Table 4:** Factors predicting the implementation of the NHP in Nepal.

Variable	Sig.	Crude Odds Ratio	95% CI for CoR [Lower–Upper]
Age	**0.000**	0.919	[0.880–0.961]
Gender (Ref: Female)			
Male	0.536	0.815	[0.426–1.557]
Profession (Ref: PHI/PHO/Pharmacist)			
Nursing staffs	0.566	0.667	[0.167–2.666]
Allied Health Workers	0.919	0.943	[0.303–2.932]
Medical Doctors	0.283	0.565	[0.199–1.604]
Mode of employment (Ref: Volunteer)			
Permanent	0.572	1.333	[0.492–3.614]
Contract	0.782	1.212	[0.311–4.730]
Temporary/part-time	0.594	1.382	[0.420–4.541]
Use of the technology in work (Ref: Rare)			
Regular use	**0.001**	5.448	[1.988–14.926]
Occasional use	0.762	1.202	[0.365–3.956]
Access to healthcare resources (Ref: Insufficient)			
Moderate	0.336	1.376	[0.718–2.636]
Received training on NHP (Ref: No)			
Yes	0.543	0.761	[0.315–1.837]
Local authorities support NHP implementation. (Ref: No)			
Yes	0.427	1.621	[0.493–5.334]
Year of experience in the health sector	**0.002**	0.934	[0.895–0.975]

*p* < 0.05. Bold values indicate statistically significant associations.

Mode of employment was not significantly associated with the Implementation of the NHP. Permanent employees had a CoR of 1.333 (95% CI [0.492–3.614], *p* = 0.572), contract employees had a CoR of 1.212 (95% CI [0.311–4.730], *p* = 0.782), and temporary/part-time employees had a CoR of 1.382 (95% CI [0.420–4.541], *p* = 0.594) compared to volunteers. Use of technology in work showed a significant association for regular use (CoR = 5.448, 95% CI [1.988–14.926], *p* = 0.001), while occasional use was not significant (CoR = 1.202, 95% CI [0.365–3.956], *p* = 0.762). Access to healthcare resources was not significantly associated with implementing the NHP, with moderate access showing a CoR of 1.376 (95% CI [0.718–2.636], *p* = 0.336). Training on NHP did not yield a significant association, with a CoR of 0.761 (95% CI [0.315–1.837], *p* = 0.543). Support from local authorities for NHP implementation was also not significant, with a CoR of 1.621 (95% CI [0.493–5.334], *p* = 0.427). Years of experience in the health sector significantly predicted the Implementation of the NHP (CoR = 0.934, 95% CI [0.895–0.975], *p* = 0.002). These results identify significant and non-significant associations among the variables examined.

## Discussion

This research evaluates the implementation of Nepal’s NHP locally, focusing on challenges within the federal system. It examines coordination between different government levels and explores the perspectives of healthcare providers.

Understanding the awareness and comprehension levels of the NHP among local health workers is crucial for effective policy implementation and healthcare delivery. Regarding awareness of the NHP, the current study indicates a moderate level of familiarity among health workers of policy. Moreover, the diverse sources through which health workers receive information about the policy, including training sessions, official documents, workshops, and colleagues, emphasize the importance of a multi-channel approach to disseminating information and promoting awareness. Our findings highlight the need for targeted interventions, such as enhanced training programs and communication strategies, to strengthen the NHP implementation and enhance its impact on healthcare delivery. For this, the timely and effective use of a sufficient budget is critical for successfully implementing health policy (25).

The challenges in NHP implementation are evident in Nepal, with perceived difficulties in accessing healthcare services, managing medicines, and addressing the profit orientation of the health sector. Likewise, this found obstacles to providing free, quality basic health services due to inadequate infrastructure and limited access to skilled professionals. Studies underscored similar challenges, including unequal healthcare distribution, poor infrastructure, insufficient essential drugs, unregulated private providers, inadequate health budget allocation, and rural human resource (17) retention issues also found that only 61.8% of Nepalese households have timely access to healthcare facilities, underscoring the need for urgent population-level interventions (26), as there is far less than the World Health Organization’s (WHO) recommendation of 2.3 doctors, nurses, and midwives per 1,000 people in Nepal (27). Similarly, a study in China discovered that the increasing inequity in subnational public expenditure suggests that subnational-level resources and responsibilities were not well aligned with national priorities (28).

Despite challenges, the present study findings also point toward opportunities for improvement, such as increased collaboration among different levels of governance, raising public awareness, and leveraging technological advancements. Similarly, the federal environment of Nepal has facilitated direct collaboration between local governments and constituents, enabling better funding and evidence-based planning tailored to community needs, thus increasing financial support for health initiatives (4, 29). The Nepal Health Policy (NHP) provides a broad vision and framework for the healthcare system, focusing on long-term goals like equity, accessibility, and quality. In contrast, the Nepal Health Sector Strategy (NHSS) 2015–2020 was an action-oriented plan with specific objectives but faced challenges due to poor coordination among policy actors, resulting in inefficiencies and inconsistencies in service delivery (15). Similarly, Wasti et al. (17) a study conducted in Nepal, also reported that challenges like poor coordination, delayed funds, staff maldistribution, procurement issues, and inadequate monitoring underscore systemic deficiencies hampering healthcare delivery in Nepal.

This study reveals important insights into implementing the NHP. While 45.8% of respondents agreed that the NHP objectives were clear and 56.6% found the goals understandable, concerns about financial resources were prominent. The study reveals a gap in funding for the NHP, with 65.1% of respondents feeling funds are insufficient and 58.5% questioning the adequacy of public health spending. This highlights the need for sustainable financing. Studies highlight that diversifying funding sources through international aid and public-private partnerships and increasing the public health budget to support the NHP’s goals effectively (30).

Political factors also showed mixed results. While 65% saw political influence as positive, 68.1% identified political interference as a hindrance, suggesting that political stability and governance reforms are essential. Political hindrance in Nepal’s health policy implementation stems from political instability, frequent leadership changes, corruption, and mismanagement of resources. Political polarization and lack of consensus among parties also prevent unified action, while weak governance structures allow political interference to undermine the effectiveness of public health programs (31).

Human resources were another key concern, with 45.8% agreeing there is a shortage of competent personnel, and 13.8% disagreed that personnel were adequately prepared. This underscores the need for targeted training and capacity-building. Nepal’s shortage of competent healthcare personnel is due to limited training opportunities, brain drain, insufficient professional development, low compensation, and unequal distribution, with urban areas having more healthcare workers than rural regions (32).

Regarding Factors Predicting the Implementation of the NHP in Nepal, Age was significantly associated with a positive attitude toward the implementation of the NHP in Nepal, with an odds ratio (OR) of 0.919 (95% CI, 0.880–0.961, *p* < 0.001). The association between age and a positive perception toward implementing Nepal’s NHP can be attributed to several factors. Younger individuals may exhibit a more progressive mindset and greater adaptability to change, fostering positive attitudes toward new policies (33). They are often more exposed to contemporary public health information and are more likely to embrace innovations and reforms in the healthcare system (34).

The use of technology in work was significantly associated with positive outcomes in NHP implementation. Regular use of technology was associated with a significantly higher likelihood (OR: 5.448, 95% CI: 1.988–14.926, *p* = 0.001) than rare use. However, occasional use was not significantly associated (OR: 1.202, 95% CI: 0.365–3.956, *p* = 0.762). The significant association between regular use of technology and positive outcomes may be its ability to streamline workflows, enhance efficiency, and provide better access to information (35). Regular users are likely more skilled in utilizing technological tools, enabling them to adapt effectively to modern work demands and policy implementations (36).

In contrast, occasional users may lack proficiency or consistent exposure, limiting their ability to leverage technology’s benefits fully. This could explain the lack of a significant association in this group. Frequent engagement with technology fosters familiarity, confidence, and a greater capacity to achieve favorable outcomes in professional settings. Years of experience in the health sector were significantly associated with the Implementation of the NHP (OR: 0.934, 95% CI: 0.895–0.975, *p* = 0.002). This indicates that for each additional year of experience, the odds of supporting the implementation of the NHP decrease. The inverse association may reflect that individuals with more years of experience tend to rely on established practices and may be less receptive to adopting new approaches or policies (34). In contrast, those with less experience may exhibit greater openness to innovation and adaptability in implementing new health sector strategies. This highlights the need for targeted interventions, such as tailored training programs and awareness campaigns, to engage experienced healthcare professionals and encourage their participation in adopting and implementing new policies effectively (37, 38).

### Strengths of the study

This study is the first of its kind in Nepal, addressing a critical gap in understanding the implementation of the NHP within the federal context. It employed a robust quantitative design, incorporating 166 health workers from various health offices, including medical doctors, nursing staff, allied health professionals, pharmacists, and public health personnel. This diversity ensures a comprehensive representation of healthcare perspectives. The structured questionnaire, carefully developed through a literature review and expert consultations, enhanced the content validity and reliability of the findings. Combining Likert-scale items with detailed demographic analysis offers valuable insights into the challenges, opportunities, and factors influencing NHP implementation at the local level, emphasizing the need for such research in Nepal’s federal framework.

### Limitations of the study

The study faced several methodological constraints that may affect the generalizability of its findings. The focus on health workers solely within the Lumbini province may not fully represent the perspectives of healthcare workers in other regions of Nepal, particularly rural areas with different healthcare challenges.

### Implications of the study

The findings significantly affect policy and practice in Nepal’s healthcare system. First, it highlights the critical need to strengthen health workers’ awareness and understanding of the NHP through targeted training programs and enhanced communication channels. Second, the identified challenges in healthcare access, insurance efficiency, and resource allocation underscore the necessity for systemic reforms and increased investment in healthcare infrastructure, particularly in underserved areas. Finally, the mixed perceptions about resource distribution, political interference, and personnel competency emphasize the importance of developing multi-stakeholder approaches to address these challenges, potentially through improved inter-governmental coordination and sustainable funding mechanisms. These implications are particularly relevant for policymakers and healthcare administrators working to enhance the effectiveness of NHP implementation locally.

## Conclusion

In conclusion, this study highlights the challenges and opportunities of implementation. Despite moderate awareness among health workers, significant barriers such as financial constraints, inadequate human resources, political interference, and infrastructure limitations persist. However, the federal system offers the potential for improved coordination and local-level collaboration, which can enhance policy effectiveness. Technology use, age, and experience influence positive attitudes toward NHP implementation, underscoring the need for targeted interventions, capacity-building, and sustainable funding. Addressing these systemic challenges, promoting greater political stability, and leveraging technological advancements while ensuring a more equitable distribution of healthcare resources across the country is crucial to achieving the policy’s goals.

## Data Availability

The original contributions presented in the study are included in the article/Supplementary material, further inquiries can be directed to the corresponding author.
