# Correction: National Health Policy and factors predicting its implementation at the local level in Nepal: an exploratory cross-sectional study

**DOI:** 10.3389/fpubh.2026.1761228

**Published:** 2026-02-03

**Authors:** Dipendra Khadka, Zhang Xinyi, Zhang Mengjie, Rajan Bhusal, Chichen Zhang

**Affiliations:** 1 School of Public Health, Southern Medical University, Guangzhou, Guangdong, China; 2 School of Health Management, Southern Medical University, Guangzhou, Guangdong, China; 3 Key Laboratory of Philosophy and Social Sciences of Colleges and Universities in Guangdong Province for Collaborative Innovation of Health Management Policy and Precision Health Service, Guangzhou, Guangdong, China; 4 School of International Education, Southern Medical University, Guangzhou, Guangdong, China; 5 Hospital and Rehabilitation Centre for the disabled children (HRDC), Banepa, Nepal

**Keywords:** national health, healthcare workers, health policy, Nepal, public health

Affiliations and superscript numbers were erroneously written as:

Dipendra Khadka^1, 2, 3*^, Zhang Xinyi^1, 2^, Zhang Mengjie^1, 2^, Rajan Bhusal^4^, Chichen Zhang^1, 2^

1 School of Health Management, Southern Medical University, Guangzhou, China

2 Key Laboratory of Philosophy and Social Sciences of Colleges and Universities in Guangdong Province for Collaborative Innovation of Health Management Policy and Precision Health Service, Guangzhou, China

3 School of International Education, Southern Medical University, Guangzhou, China

4 Hospital and Rehabilitation Centre for the Disabled Children (HRDC), Banepa, Nepal

The correct affiliation list with correct superscripts for the authors are as follows:

Dipendra Khadka^1, 2, 3, 4^, Zhang Xinyi^2, 3^, Zhang Mengjie^2, 3^, Rajan Bhusal^5^, Chichen Zhang^1, 2, 3*^

1 School of Public Health, Southern Medical University, Guangzhou, Guangdong, China

2 School of Health Management, Southern Medical University, Guangzhou, Guangdong, China

3 Key Laboratory of Philosophy and Social Sciences of Colleges and Universities in Guangdong Province for Collaborative Innovation of Health Management Policy and Precision Health Service, Guangzhou, Guangdong, China

4 School of International Education, Southern Medical University, Guangzhou, Guangdong, China

5 Hospital and Rehabilitation Centre for the disabled children (HRDC), Banepa, Nepal

Author [Dipendra Khadka] was erroneously assigned as corresponding author. The correct corresponding author is [Chichen Zhang].

The original version of this article has been updated.

