# Supplementary material for: National Health Policy and factors predicting its implementation at the local level in Nepal: an exploratory cross-sectional study
Source: Front Public Health. 2025 Jul 4;13:1592213. doi: 10.3389/fpubh.2025.1592213 (PMC12271155; doi:10.3389/fpubh.2025.1592213)
Supplement: Supplementary file 2 [file Table_2.docx]

**Questionnaire for Study on Awareness, Challenges, and Opportunities in Implementing the National Health Policy (NHP)**

Form No.:

Consent Form:

I, voluntarily agree to participate in this study titled “National health policy and its

implementation in local level of Nepal”. I understand that my involvement will include

completing a questionnaire aimed at the level of awareness and comprehension of the National

Health Policy among health workers at the local level, identify challenges and opportunities for

its implementation in Nepalese communities, and explore factors influencing its implementation.

I acknowledge that my participation is entirely voluntary, and I have the right to withdraw from

the study at any time without consequence. I am aware that the information collected will be

used for research purposes only and will be kept confidential. I have had the opportunity to ask

any questions and have received satisfactory answers. By proceeding with this questionnaire, I

give my informed consent to participate in this study.

Signature: ____________________

Date: ____________________

**Questionnaire**

**Section 1: Demographic and Socioeconomic Characteristics**

1. **Age Group** (Please tick one):
   - Less than 30 years
   - 30-40 years
   - 40-50 years
   - Above 50 years
2. **Sex** (Please tick one):
   - Male
   - Female
3. **Type of Health Worker** (Please tick one):
   - Medical Doctor
   - Nursing Staff
   - Allied Health Worker (e.g., HA, ANM, AHW, Lab Technician)
   - Pharmacist
   - PHI/PHO
4. **Years of Experience** (Please tick one):
   - 0-5 years
   - 5-10 years
   - 10-15 years
   - More than 15 years
5. **Mode of Employment** (Please tick one):
   - Permanent
   - Contract
   - Temporary/Part-time
   - Volunteer
6. **Use of Technology in Work** (Please tick one):
   - Regular Use
   - Occasional Use
   - Rare
7. **Access to Healthcare Resources** (Please tick one):
   - Moderate
   - Insufficient

**Section 2: Awareness of the National Health Policy (NHP)**

1. How familiar are you with the National Health Policy (NHP)?
   - Not familiar at all
   - Not very familiar
   - Neutral
   - Somewhat familiar
   - Very familiar
2. How well do you understand the key objectives and components of the NHP?
   - Not well at all
   - Not very well
   - Neutral
   - Moderately well
   - Very well
3. How do you primarily receive information about the NHP? (Tick all that apply):
   - Training Sessions
   - Official Documents
   - Workshops or Seminars
   - Colleagues or Superiors
   - Other: __________________
4. Are you aware of the strategies and initiatives of the NHP at the local level?
   - Very aware
   - Somewhat aware
   - Neutral
   - Not very aware
   - Not aware at all
5. Do you feel the need for additional training or resources to understand the NHP better?
   - Strongly agree
   - Agree
   - Neutral
   - Disagree
   - Strongly disagree

**Section 3: Challenges in Implementing the NHP**

1. What are the main obstacles to providing quality health services? (Tick all that apply):
   - Lack of financial resources
   - Limited access to skilled healthcare professionals
   - Inadequate infrastructure and facilities
   - High out-of-pocket expenditures
   - Inefficient health insurance policies
   - Lack of awareness among the public
2. How do you rate the support from local authorities for NHP implementation?
   - Very High
   - High
   - Moderate
   - Low
   - Very Low
3. Do you agree that there is a shortage of competent health personnel to execute the NHP effectively?
   - Strongly agree
   - Agree
   - Neutral
   - Disagree
   - Strongly disagree

**Section 4: Opportunities for Enhancing NHP Implementation**

1. How do you rate the opportunities to increase public awareness of health-related issues?
   - Excellent Opportunities
   - Good Opportunities
   - Some Opportunities
   - Limited Opportunities
   - No Opportunities
2. To what extent do advancements in technology, drugs, and equipment influence NHP implementation?
   - Extremely
   - Very Much
   - Moderately
   - Slightly
   - Not at All
3. How do you rate the emphasis of health policies and programs on healthcare quality management?
   - Extremely
   - Very Much
   - Moderately
   - Slightly
   - Not at All

### Section 5:Factors Influencing the Implementation of the NHP

Please indicate your level of agreement with the following statements regarding factors influencing the implementation of the National Health Policy (NHP).

1. The objectives outlined in the National Health Policy are clearly communicated.
   - Strongly agree
   - Agree
   - Neutral
   - Disagree
   - Strongly disagree
2. The goals of the National Health Policy are easily understandable.
   - Strongly agree
   - Agree
   - Neutral
   - Disagree
   - Strongly disagree
3. The financial resources allocated for NHP implementation are sufficient.
   - Strongly agree
   - Agree
   - Neutral
   - Disagree
   - Strongly disagree
4. There is a lack of clarity in the distribution of financial resources for the NHP.
   - Strongly agree
   - Agree
   - Neutral
   - Disagree
   - Strongly disagree
5. Political influences positively contribute to the effective implementation of the NHP.
   - Strongly agree
   - Agree
   - Neutral
   - Disagree
   - Strongly disagree
6. Political interference negatively impacts the successful execution of the NHP.
   - Strongly agree
   - Agree
   - Neutral
   - Disagree
   - Strongly disagree
7. Health personnel are well-equipped and competent in implementing NHP strategies.
   - Strongly agree
   - Agree
   - Neutral
   - Disagree
   - Strongly disagree
8. There is a shortage of competent health personnel to effectively execute the NHP.
   - Strongly agree
   - Agree
   - Neutral
   - Disagree
   - Strongly disagree
9. Public health expenses for the NHP are appropriately allocated to address healthcare needs.
   - Strongly agree
   - Agree
   - Neutral
   - Disagree
   - Strongly disagree
10. The current allocation of public health expenses is inadequate for the comprehensive implementation of the NHP.
    - Strongly agree
    - Agree
    - Neutral
    - Disagree
    - Strongly disagree
11. There are ample resources, both human and material, to support the successful implementation of the NHP.
    - Strongly agree
    - Agree
    - Neutral
    - Disagree
    - Strongly disagree
12. Resources, both human and material, are insufficient to meet the requirements of NHP initiatives.
    - Strongly agree
    - Agree
    - Neutral
    - Disagree
    - Strongly disagree
13. The organization provides strong support for the implementation of NHP initiatives.
    - Strongly agree
    - Agree
    - Neutral
    - Disagree
    - Strongly disagree
14. Individual health workers actively engage and contribute to the successful execution of the NHP.
    - Strongly agree
    - Agree
    - Neutral
    - Disagree
    - Strongly disagree

**Section 5: Feedback and Suggestions**

1. What changes do you suggest for improving the implementation of the NHP at the local level?

Any additional comments or observations?

THANK YOU FOR THE PARTICIPATION
