# Supplementary material for: National Health Policy and factors predicting its implementation at the local level in Nepal: an exploratory cross-sectional study
Source: Front Public Health. 2025 Jul 4;13:1592213. doi: 10.3389/fpubh.2025.1592213 (PMC12271155; doi:10.3389/fpubh.2025.1592213)
Supplement: Supplementary file 3 [file Table_3.docx]

***S*ocioeconomic characteristics of Study Participants**

| **Demographic Characteristics** | **Frequency (N)** | **Percent (%)** |
| --- | --- | --- |
| **Age group** |  |  |
| Less than 30 years | 54 | 32.5 |
| 30-40 years | 72 | 43.4 |
| 40-50 years | 36 | 21.7 |
| Above 50 years | 4 | 2.4 |
| **Sex** |  |  |
| Male | 64 | 38.6 |
| Female | 102 | 61.4 |
| **Types of the health workers** |  |  |
| Medical doctors | 16 | 9.6 |
| Nursing Staff | 47 | 28.3 |
| Allied health workers (HA/ANM/AHW/Lab technician/Lab Assistant/Radiographer/Radiologist) | 82 | 49.4 |
| Pharmacist | 11 | 6.6 |
| PHI/PHO | 10 | 6.0 |
| **Experiences of Health workers (in years)** |  |  |
| (0-5) years | 39 | 23.5 |
| (5-10) years | 55 | 33.1 |
| (10-15) years | 27 | 16.3 |
| More than 15 years | 45 | 27.1 |
| **Mode of employment** |  |  |
| Permanent | 102 | 61.4 |
| Contract | 16 | 9.6 |
| Temporary/Part-time | 29 | 17.5 |
| Volunteer | 19 | 11.4 |
| **Use of technology in work** |  |  |
| Regular use | 118 | 71.1 |
| Occasional use | 28 | 16.9 |
| Rare | 20 | 12.0 |
| **Access to healthcare resources** |  |  |
| Moderate | 69 | 41.6 |
| Insufficient | 97 | 58.4 |
| **Received training on the National Health Program (NHP)** |  |  |
| Yes | 24 | 14.5 |
| No | 142 | 85.5 |
| **Local government authorities support NHP implementation.** |  |  |
| Yes | 15 | 9.0 |
| No | 151 | 91.0 |
| **Source of information about NHP** |  |  |
| Training sessions | 54 | 32.5 |
| Official documents | 61 | 36.7 |
| Workshops or seminars | 17 | 10.2 |
| Colleagues/superiors | 21 | 12.7 |
| Other | 13 | 7.8 |

**Main obstacles to providing free, quality basic health services in the local community**

| **Obstacles** | **Frequency (N)** | **Percent (%)** |
| --- | --- | --- |
| **Lack of financial resources** |  |  |
| No | 76 | 45.8% |
| Yes | 90 | 54.2% |
| **Limited access to skilled healthcare professionals** |  |  |
| No | 73 | 44.0% |
| Yes | 93 | 56.0% |
| **Inadequate infrastructure and facilities** |  |  |
| No | 63 | 38.0% |
| Yes | 103 | 62.0% |
| **High out-of-pocket expenditure for healthcare** |  |  |
| No | 117 | 70.5% |
| Yes | 49 | 29.5% |
| **Inefficient health insurance policies** |  |  |
| No | 94 | 56.6% |
| Yes | 72 | 43.4% |
| **Lack of awareness** |  |  |
| No | 160 | 97.0% |
| Yes | 5 | 3.0% |
